# Do Behavioral Foraging Responses of Prey to Predators Function Similarly in Restored and Pristine Foodwebs?

**DOI:** 10.1371/journal.pone.0032390

**Published:** 2012-03-05

**Authors:** Elizabeth M. P. Madin, Steven D. Gaines, Joshua S. Madin, Anne-Katrin Link, Peggy J. Lubchenco, Rebecca L. Selden, Robert R. Warner

**Affiliations:** 1 Department of Ecology, Evolution and Marine Biology, University of California Santa Barbara, Santa Barbara, California, United States of America; 2 Department of Biological Sciences, Macquarie University, Sydney, New South Wales, Australia; 3 Marine Science Institute, University of California Santa Barbara, Santa Barbara, California, United States of America; 4 Bren School of Environmental Science and Management, University of California Santa Barbara, Santa Barbara, California, United States of America; University of Hull, United Kingdom

## Abstract

Efforts to restore top predators in human-altered systems raise the question of whether rebounds in predator populations are sufficient to restore pristine foodweb dynamics. Ocean ecosystems provide an ideal system to test this question. Removal of fishing in marine reserves often reverses declines in predator densities and size. However, whether this leads to restoration of key functional characteristics of foodwebs, especially prey foraging behavior, is unclear. The question of whether restored and pristine foodwebs function similarly is nonetheless critically important for management and restoration efforts. We explored this question in light of one important determinant of ecosystem function and structure – herbivorous prey foraging behavior. We compared these responses for two functionally distinct herbivorous prey fishes (the damselfish *Plectroglyphidodon dickii* and the parrotfish *Chlorurus sordidus*) within pairs of coral reefs in pristine and restored ecosystems in two regions of these species' biogeographic ranges, allowing us to quantify the magnitude and temporal scale of this key ecosystem variable's recovery. We demonstrate that restoration of top predator abundances also restored prey foraging excursion behaviors to a condition closely resembling those of a pristine ecosystem. Increased understanding of behavioral aspects of ecosystem change will greatly improve our ability to predict the cascading consequences of conservation tools aimed at ecological restoration, such as marine reserves.

## Introduction

Conservation and management actions designed to restore ecosystems to their historical states have typically focused on recovery of the directly disturbed aspects of the system, such as target stocks of fisheries. In recent years, however, a shift in focus has led to increased interest in understanding and predicting whole-ecosystem responses to such management measures in accordance with the principles of ecosystem-based management (EBM). In general, human alterations of food webs in the marine environment often disproportionately affect higher trophic levels [1], [2]. Changes in abundances of upper-level consumers (e.g., predators) are known to alter both the structure and function of lower trophic levels, for example through both density- and behaviorally-mediated trophic cascades [3]–[6]. We now know that removing human disturbances can in many cases restore predator populations. However, we do not know whether rebounds in predator populations are sufficient to allow key functional characteristics of foodwebs, such as prey foraging behavior, to return to pre-disturbance levels. One way to answer this question is to compare systems with three different historical trajectories, namely those that have been largely undisturbed, those that are declining due to human disturbance, and those that are recovering following the removal of human diturbance (e.g., protected areas). Resolving this issue is key to evaluating the success of any conservation or management action designed to restore a degraded system to its pristine, or pre-disturbance, state.

Fishing in marine systems is analogous to a large-scale, long-term, uncontrolled ‘natural experiment’ through which dynamics of both declining and recovering ecosystems can be observed. In many parts of the world, fishing tends to focus disproportionately on larger-bodied, predatory fishes, with fished reefs generally exhibiting reduced predator densities and biomass [7]. This global-scale ‘experiment’ can be used to help us clarify the direct and indirect effects of predator loss and, following its cessation, predator recovery. Globally, most reefs have experienced at least a moderate degree of historical fishing pressure [2], but an exceedingly rare few have escaped significant fishing because of their isolation. In a few situations, these *de facto* refuges have biogeographically comparable fished reefs that can help us estimate the consequences of predator removal on ecosystem dynamics. The northern Line Islands of the Eastern Indo-Pacific provide one important comparison of this type [8], [9].

In recent decades, parallel gradients in fishing intensity among reefs have been created through the establishment of no-take marine reserves around the globe (e.g., Australia's Great Barrier Reef). However, unlike the pristine reef ecosystems surrounding some of the Line Islands, which have a history of little or no fishing, the marine reserves of the Great Barrier Reef eliminate fishing from reefs that have historically been fished. As predators recover in the absence of fishing on these reefs, the dynamics of recovering ecosystems can be compared to the far rarer historically-intact systems. The unresolved question is how much of the pristine, or pre-disturbance, ecosystem function can be recovered by ecosystem restoration. In terms of the behavioral pathway of ecosystem change, if an ecosystem follows a similar trajectory during recovery as it does during decline, the behavioral responses of prey following predator recovery should mimic the behavior of prey in pristine reefs with natural, baseline levels of predation risk.

The mechanisms underlying the indirect effects of predator removal through fishing are generally not well understood, but several mechanisms have recently been reported. Prey behavioral responses to altered predation risk have been shown to play a key role in marine ecosystems [6], [10], [11], including coral reefs [12], but the question remains as to how ubiquitous this mechanism may be. Madin et al. [13] recently demonstrated that prey foraging behavior is dramatically affected by the loss of predators due to fishing, and Madin et al. [14] subsequently showed that these responses can cascade through the ecosystem to affect macroalgal distribution. These studies reveal that prey foraging behavioral dynamics, which are known to mediate top-down effects (e.g., predator loss) in other ecosystem types [5], [15], [16], can play a crucial role in mediating the effects of fishing in coral reef systems [12]. However, these focus solely on the effects of predator *loss* from fishing, whereas the effects of predator *recovery* on prey behavior have yet to be explored. Understanding such effects will be critical as efforts to restore ecosystems to their former states, for example through marine reserves, are increasingly implemented worldwide.

**Figure 1:**
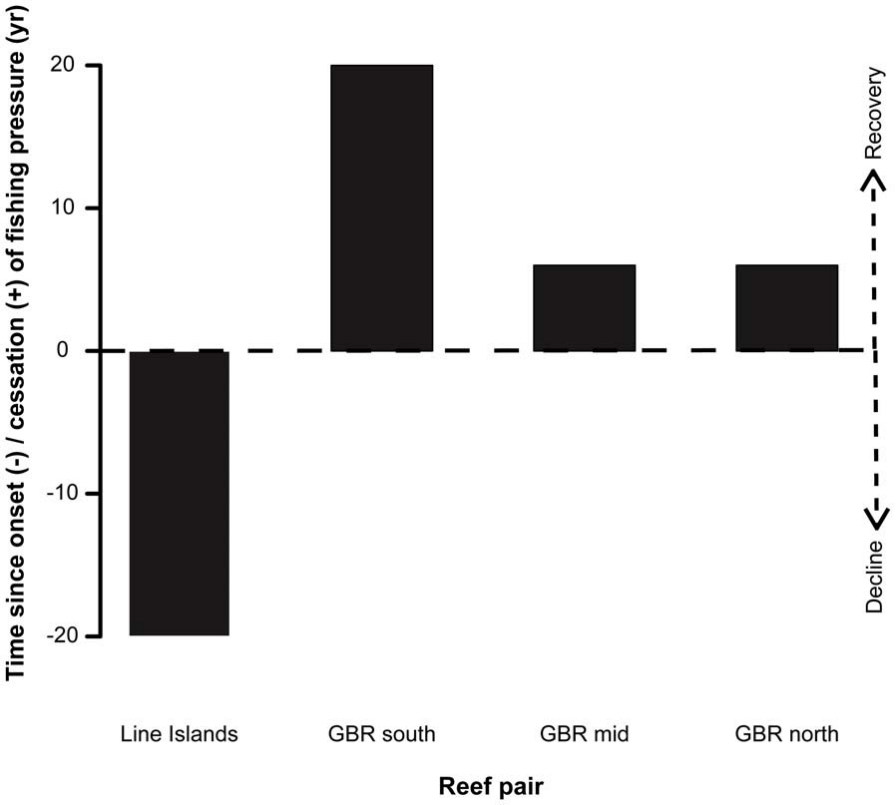
History of fishing pressure on reef fishes for reef pairs used in this study. X-axis indicates reef pair. Y-axis indicates the amount of time elapsed since either onset (Line Islands) or cessation (GBR) of fishing in each of the reef pairs, in years. Negative values therefore represent the length of time that the Eastern Indo-Pacific (Line Islands) fished site has been exploited (i.e., indicated as “decline” by dashed arrow on right-hand y-axis), whereas positive values represent time since cessation of fishing at protected Central Indo-Pacific (GBR) sites (i.e., as indicated by the “recovery” side of dashed arrow). “Decline” and “recovery” refer to the presumed trajectory of reefs' exploited fish populations due to fishing pressure, not to structural changes in reefs.

**Figure 2:**
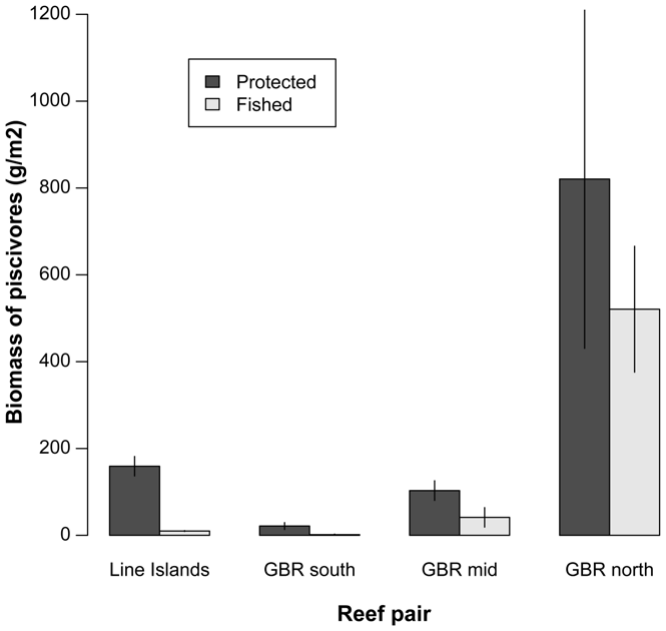
Biomass of piscivorous fishes. Piscivorous fish biomass (per unit reef area) at reefs used in this study. Bars are means (±SE).

Here we use a suite of paired fished and unfished reefs to explore the restoration of one key aspect of ecosystem functioning: prey foraging behavior. Specifically, we do so by asking whether differences in prey foraging behavior between a near-pristine system within a marine reserve and its heavily-fished counterpart that had previously been a de facto reserve (due to its geographic isolation) in the Line Islands are similar to differences in prey behavior between reef systems in which protected predator populations have recovered relative to unprotected, fished analogues in the Great Barrier Reef. Figure 1 describes the history of fishing for the suite of reef pairs used in terms of the amount of time elapsed since the onset or cessation of fishing within each pair. Pairs of adjacent reefs under different fishing regimes situated within each of these realms have dramatically different average piscivore biomass as a result of their different fishing histories and regulations (Fig. 2). We show for two functionally-distinct herbivorous fish species that removal of fishing pressure, and the resultant recovery of top predators, leads to restoration of prey foraging behavior that is comparable to those of historically pristine systems, and in particular that this restoration can occur within only a few years of fishing closures.

## Results

The two major areas surveyed differ both in their biogeographic locations (the GBR is within the Central Indo-Pacific, whereas the Line Islands are situated in the Eastern Indo-Pacific) and in their respective histories of human fishing pressure (Fig. 1). In particular, the paired reefs used in the GBR differ in that one reef has been afforded protection from fishing following historical fishing and one reef continues to be fished, whereas the reef pair used in the Line Islands differs in that one reef has never been fished and one has been fished only in recent years.

Within all reef pairs, mean biomass density of piscivorous fishes is greater on the unfished than the fished reefs (Fig. 2; *F*_1,202_ = 42.84; *P*<0.001) and also differs significantly among the different regions of the reef pairs (*F*_3,202_ = 25.66; *P*<0.001). The protection status (fished vs. unfished)×region interaction is also significant (*F*_3,202_ = 3.73; *P*<0.05), a likely result of the decreasing proportional differences in relative piscivore biomass density between fished/unfished reefs with increasing distance from human population centers. The only reef pair with a non-significant difference in mean piscivore biomass is the most remote Central Indo-Pacific reef pair, GBR north, a likely result of the relatively light fishing pressure on the fished reef in this pair. While commercial fishing does occur in this region, both fishing effort and catch tend to be lower overall than in the more southerly regions used in this study [17]. The far offshore location of these reefs further decreases the likelihood of the fished reef from this pair being fished, resulting in a ‘fished’ reef that could be considered to be approaching a de facto reserve itself.

**Figure 3:**
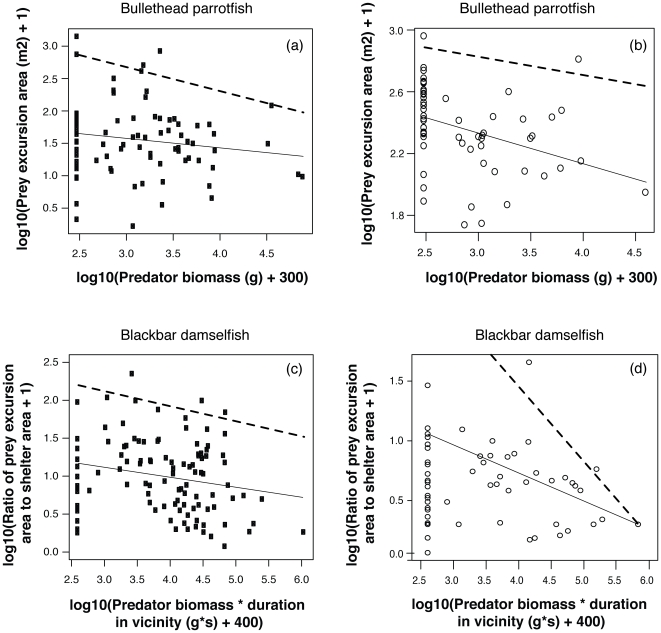
Prey excursion size and rate of movement in relation to acute predation risk for *C. sordidus* and *P. dickii*. Left-hand panels (a, c) show data from the Eastern Indo-Pacific (Line Islands); right-hand panels (b, d) are from the Central Indo-Pacific (GBR). Lines show best-fit upper 95% prediction intervals (dashed) and linear regressions (solid) based on a negative log-likelihood optimization function. Points are values for individual prey where predation risk is measured by predator biomass for *C. sordidus* and predator (biomass×duration) for *P. dickii*. Eastern Indo-Pacific (right-hand) panels are reproduced with permission from Madin et al. [13].

Both prey species observed, the parrotfish *C. sordidus* and the damselfish *P. dickii*, demonstrate the same qualitative pattern of declining excursion size as acute predation risk increases (Fig. 3). The effect of predation risk upon prey excursion size is most effectively measured as the maximum, rather than the mean, size of excursions that prey are willing to take. This is because predation risk should limit maximum, but not minimum, excursion sizes of prey [13], allowing prey under low risk to take either long or short excursions from shelter, but limiting those under high risk to generally only taking shorter excursions. We therefore used the upper bound of the prediction interval, rather than standard regression through the mean, to assess the effect of predation risk on prey behaviour. Slopes of all upper prediction intervals and regressions were significant at the α = 0.05 level with the exceptions of *C. sordidus*' regression slope in the Eastern Indo-Pacific, which was marginally significant (*P* = 0.06), and the upper bound of this species' relationship in the Central Indo-Pacific (*P* = 0.33). Acute risk is a metric of the actual predation risk to which a focal prey individual was exposed during the observation period. Therefore, the patterns shown in Fig. 3 are not based upon differences within particular reef pairs, but rather are reflective of the general trend among all fishes observed at all reefs.

**Figure 4:**
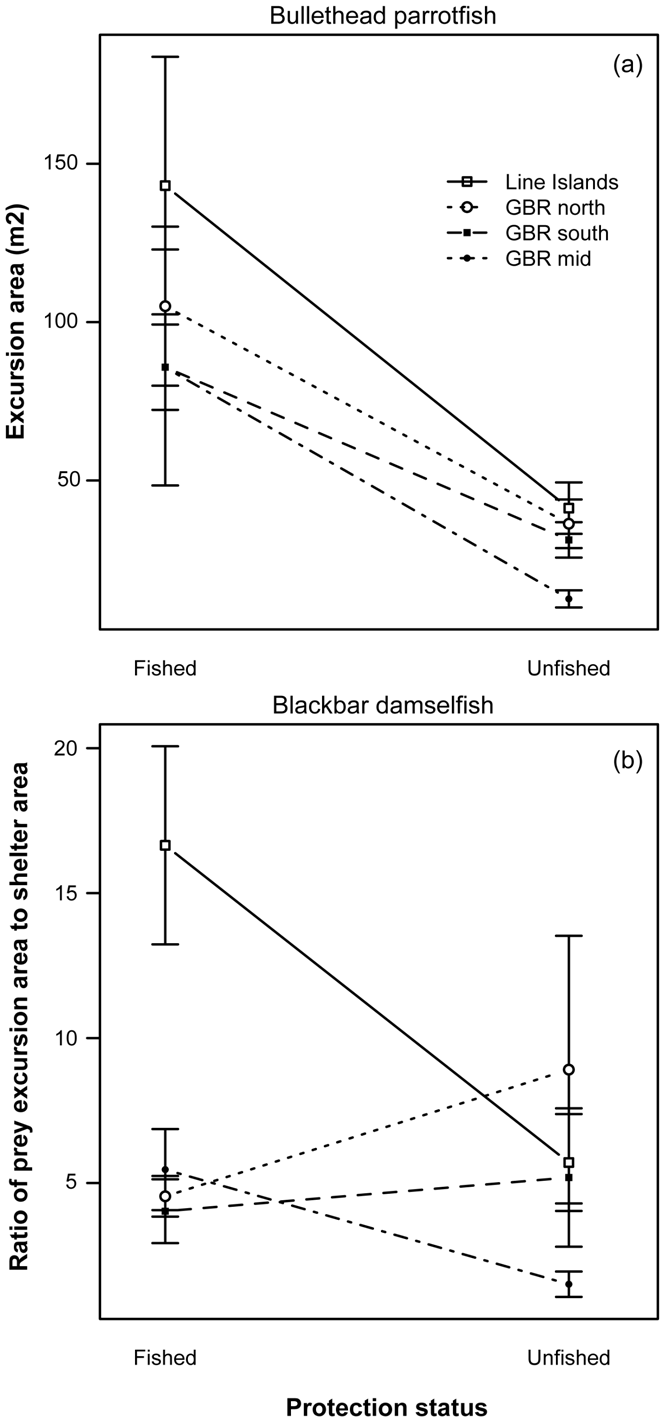
Prey excursion size in relation to protection status. Upper panel (a) is bullethead parrotfish (*C. sordidus*); lower panel (b) is blackbar damselfish (*P. dickii*). Points are means (±SE).

These patterns reflect individual prey encounters with individual predators (i.e., acute risk). Are the same patterns observed when comparing average responses over many prey individuals between fished and unfished reef pairs (i.e., chronic risk)? Indeed, the parrotfish *C. sordidus* consistently exhibits significantly smaller excursions when faced with the greater chronic risk found at the unfished, more predator-rich reefs (Fig. 4; *F*_1,181_ = 15.30; *P*<0.001). Again, this pattern holds true regardless of whether differences in piscivore biomass were due to decline versus recovery and those in different biogeographic areas.

**Figure 5:**
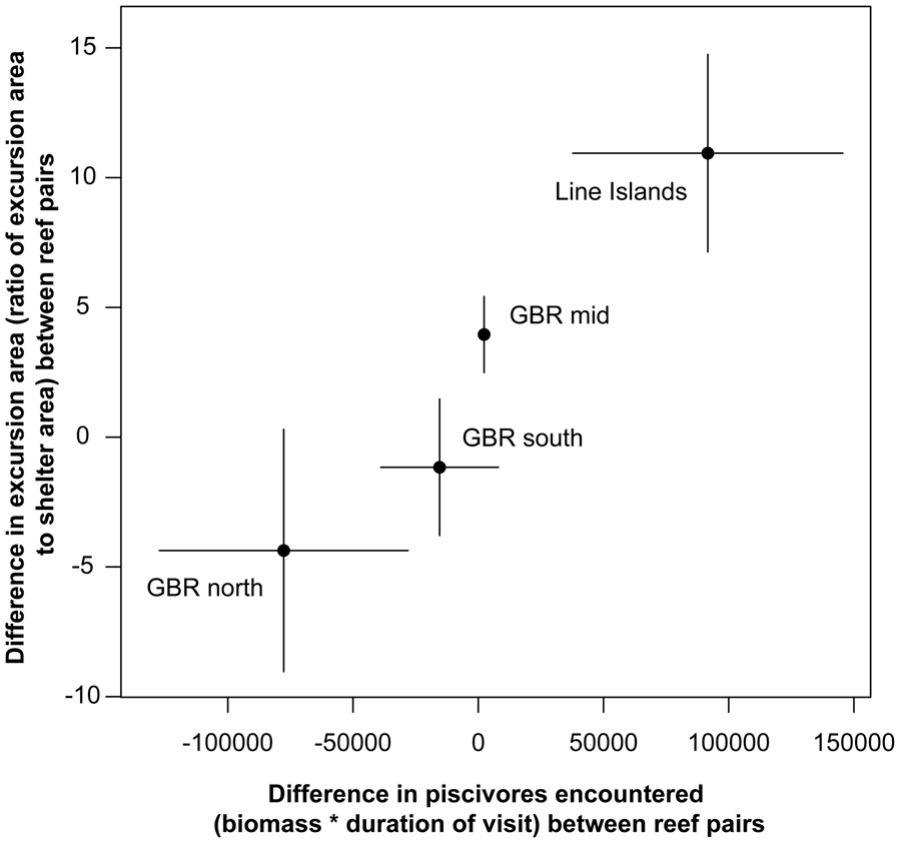
Differences in prey (*P. dickii*) movement in relation to differences in piscivorous fishes encountered between reef pairs. Points (±SE) represent differences in both independent and dependent variables between paired reefs in each of the four regions included in the study. Note that differences were calculated as unfished - fished reef values for x-axis (predation risk) and fished - unfished reef values for y-axis (prey behavior) for the reason outlined in *Materials and Methods* above.

Interestingly, the damselfish *P. dickii* does not show the same pattern when compared between fished and unfished reefs. For this species, protection status did have a significant effect on excursion size (Fig. 4; *F*_1,143_ = 7.02; *P*<0.01), but only two of the four reef pairs (Line Islands and GBR mid) exhibit the predicted decline in excursion size as a function of protection status. The remaining two reef pairs (GBR north and south) show the opposite pattern, i.e., greater excursion sizes at unfished sites with greater piscivore densities. The reason for this unexpected pattern becomes apparent upon closer inspection. Among reef pairs, this species consistently exhibits larger *differences* in average excursion size at reef pairs where they encountered larger *differences* in piscivore biomass (i.e., more and/or larger piscivores; Fig. 5). Specifically, where predator differences are positive (i.e., prey encountered more and/or larger predators in the protected than the fished reef in a pair), so are prey behaviors (i.e., prey move more in the fished than the protected reefs in a pair). This pattern demonstrates that individuals of this species are indeed responsive to predation risk at the reef scale, but that this level of risk does not match cleanly onto the protection status of reefs in these systems (Fig. S2). In this case, there is no significant effect of protection status on the biomass of piscivores encountered by this species, although the interaction between protection status and region was significant (*F*_1,113_ = 3.00; *P*<0.05).

## Discussion

1This large-scale study demonstrates that marine reserves that restore abundances of top predators can also restore an ecologically-important prey behavior, herbivore foraging excursions, closely resembling these behavioral dynamics found in a pristine ecosystem for at least two functionally distinct herbivorous species. Unlike previous studies that have explored only the effects of large-scale predator *loss* on this prey behavior [13], [14], these results document for the first time that *restoration* of predator populations can likewise result in ecologically-significant behavioral prey behavioral changes. Remarkably, prey behavioral patterns were also consistent between the two biogeographic areas surveyed, as well as between the larger parrotfish and the smaller, more vulnerable damselfish studied. Taken together with previous findings [13] of similar patterns over a broader suite of species, these patterns suggest that these prey behavioral responses to predation risk may potentially be both biogeographically and taxonomically widespread. Importantly, given the short (<5 year) duration of protection from fishing of two of the reef pairs, our findings demonstrate that in contrast to indirect effects of fishing on numerical responses of prey, which tend to exhibit time lags following predator loss or recovery [18], [19], indirect effects on prey behavioral dynamics can mirror these recoveries over closer timeframes (e.g., <5 years).

As shown in Fig. 1, and in agreement with previous studies of these regions (Line Islands [8], [9] and GBR [20]), relative differences between piscivore biomass among the reef pairs in this study were almost certainly due to the onset of fishing on previously unfished reefs (the Line Islands) and the recovery of previously fished reefs (the GBR). Given the recent nature of some of these changes in fishing pressure, our findings suggest that both piscivores and their prey have the potential to respond rapidly to protection from fishing. Previous studies of no-take reserves on the GBR have found that biomass of fished, predatory species has increased significantly within reserves, some within only a few years of protection from fishing [20]. Our results show that these increases in piscivore densities are sufficient to generate large changes in prey behaviors over these relatively short time scales. Hence, if such behavioral responses subsequently contributed (in part or in whole) to facilitating behaviorally-mediated cascades [14], we might expect these effects to occur relatively rapidly following ecosystem protection.

What is the appropriate measure of predation risk at a site? Our results suggest that it may be species-specific. For the mobile herbivore *C. sordidus*, behavioral responses to changes in predation risk are apparent at both the local and the reef-wide scales (Figs. 3 & 4a). For the site-attached herbivore/omnivore *P. dickii*, the local predation-risk environment immediately surrounding the individual appears to be the key determinant of this species' foraging excursion responses (Figs. 4b & 5). Fig. 4b shows an apparently contradictory pattern, i.e., that at some sites with higher mean predator abundances in the unfished reef of the pair (e.g., GBR south and north, although only the former is significant), this species' excursions are nonetheless larger at the unfished than the fished reef of the pair. Fig. 5 demonstrates that *P. dickii* on reefs with higher predation risk in their immediate vicinity – even if overall reef predation risk is lower – are, however, responsive to predation risk at the reef scale. The reason for this incongruence among species is unclear, but may be related to microhabitat choice of these site-attached individuals (particularly on high-risk reefs) aimed at minimizing their exposure to risk. Specifically, we propose that this disconnect between the two focal species exists because the highly site-attached *P. dickii* that rarely leaves the immediate vicinity of its home coral shelter, unlike the more mobile *C. sordidus* that moves over large areas of reef, may seek to establish its home territory in areas of lower predation risk within a given reef environment. This strategy would be expected to be employed in particular on unfished, higher-risk reefs where choosing a microhabitat to minimize predation risk would be particularly beneficial. However, as yet this hypothesis remains untested. Future studies of these or related taxa could test this premise by exploring in greater detail how microhabitat choice affects site-attached versus mobile species' exposure to predation risk. Regardless of the reason for this difference between species, our results suggest that measuring the only average behavioral responses of *P. dickii* at the reef scale (i.e., chronic risk; Fig. 2) would therefore lead to the (incorrect) conclusion that this species is behaviorally unresponsive to predation risk in terms of foraging excursions (e.g., Fig. S2). Differences in characteristic behaviors among these and other species may thus help guide selection of appropriate metrics for detecting behavioral responses under changing predator regimes.

One surprising finding of this study was that, despite having by far the highest predator biomass in both fished and unfished reefs of any of the reef pairs (Fig. 2), the GBR north reef pair did not exhibit the smallest prey excursion sizes for the bullethead parrotfish *C. sordidus* as would be expected (Fig. 4). Prey at this site had larger average excursions that prey at both the GBR south and mid sites, although this difference was only significant for the unfished GBR mid reef. It is not immediately clear why this pattern occurred, and we can only hypothesize that the high variability in predator abundances at the GBR north reefs (Fig. 2) may allow for some relaxation of the antipredator behavioural responses of this species.

Another unexpected finding of this study is the result that one of the fished, unprotected sites used in the study, that of the GBR north pair, demonstrated higher overall piscivore biomass than even the unfished reef in the Line Island pair (Fig. 2). At first glance this is surprising, because it was expected that unfished reefs would uniformly contain greater piscivore biomass than any of the fished reefs. As discussed in *Results* above, the fished reef of the GBR north pair could be considered to some extent to be a *de facto* reserve given its isolated position relative to human population centers. However, this does not offer a complete explanation for why it contains such high piscivore biomass relative to the unfished Line Islands reef. We can only hypothesize that this could potentially be explained, in part or in whole, by the generally higher species richness of Central Indo-Pacific versus Eastern Indo-Pacific reefs. To our knowledge no definitive consensus exists on the relationship between species richness and abundance across ecosystem types [21], however a number of studies across diverse ecosystems have found positive associations between these variables [21]. If this relationship is indeed positive for coral reef fishes, the greater species richness of the GBR reefs in general could help explain this finding. Unfortunately, our dataset does not allow this relationship to be explicitly tested, although future studies could make a substantial contribution to the literature by doing so.

Coral reef communities, and likewise their herbivore and predator assemblages, are inherently complex. For example, prey behavior is undoubtedly influenced by local-scale features such as habitat structure, available food resources, and the composition of both the grazer and predator assemblages [22], as well as locality-specific and historical influences on their food resources and demography [23]. Likewise, each of the study's focal (and other) prey species will almost certainly have specific suites of predators to which they will respond in specific ways. While recognizing this enormous complexity, we have nonetheless taken a broad-brush approach to examining how particular components of the reef community may change under changing human influence regimes. For example, we have included in our analyses all potential predator species – including species and individuals not likely to pose a threat to all focal ‘prey’ individuals used in the study – using the unquestionably coarse metric of total predator biomass. Similarly, we have examined behavioral changes in only two of a highly diverse herbivore assemblage, although previous findings do support the notion that the behavioral patterns we observed are common to other reef fish species [13]. Nevertheless, our broad-brush approach not only makes possible the large-scale, biogeographic comparison we present, but importantly it also renders our findings conservative. Indeed, numerous factors could lead to differing results over the geographical and historical scales we examine. Strikingly, the observed patterns show strong qualitative concordance over these scales. We do not interpret our results as suggesting that local-scale and species-specific influences are unimportant or will necessarily be overridden by a behavioral interaction driven by a generalized suite of predators. Our findings do show, however, that whatever influence these myriad factors have over prey foraging behavior, interactions between predator and prey play a pivotal role.

One simple alternative explanation of the observed reef-scale patterns (Figs. 4 and 5) of focal species' excursion sizes is competition. For both focal prey species, local population densities of competitors were quantified at all sites (Appendix S1). If local competitor density were responsible for the observed patterns, the expectation for the central place foraging, site-attached *P. dickii* would be that focal prey excursion size would be greater at reefs with higher local competitor density. This is because as local competition (defined as intraspecific group size within the focal individual's home coral colony; Appendix S1) increases, so too should the competition for resources surrounding the home coral colony and thus the need to move farther from this central place to acquire a given amount of food. However, these patterns do not match our observations. The differences in *P. dickii* excursion sizes (y-axis) presented in Fig. 5 are calculated as *excusion size_fished_ – excursion size_unfished_*. Therefore, if competition were responsible for these patterns in *P. dickii* behavior in the Central Indo-Pacific, one would expect roughly equivalent conspecific densities in both reefs of the GBR south pair and higher conspecific densities in the fished reef of the GBR mid pair and the protected reef of the GBR north pair. These patterns run counter to those observed (Fig. S3). Likewise, an explicit test of the relationship between conspecific density and *P. dickii* excursion size in the Eastern Indo-Pacific reefs revealed analogous results (described in Madin et al. [13]). The expectation for the mobile *C. sordidus* would be that excursion size would be smaller at reefs with higher local competitor density (defined as overall, interspecific benthic herbivore density; Appendix S1). This is because *C. sordidus* encounters both territorial and non-territorial benthic herbivores continually as it moves over the reef to feed, the former of which actively deter grazing within their territories and both of which contribute to crowding. Therefore, as local competition from these species increases, restriction of *C. sordidus* movement should likewise increase and excursion area should concomitantly decrease. Fig. 4 shows that for all Central Indo-Pacific reef pairs, *C. sordidus* excursions were larger in fished vs. protected reefs. One would therefore expect that, if competition were driving the observed patterns in excursion size, greater densities of benthic herbivores would be found in the protected reef of each pair. However, with the exception of the GBR south pair, in which the protected reef does exhibit marginally higher overall herbivore density, this expectation does not match observed herbivore densities (Fig. S3). In the Eastern Indo-Pacific, a similar test of the potential for competition to lead to the observed behavioral patterns concluded that this was not possible (described in Madin et al. [13]). Therefore, these patterns collectively contradict the hypothesis that local competitor density can explain the overall excursion size patterns described in this study.

Other than the impact of fishing by humans, therefore, few other natural or anthropogenic factors could potentially explain the patterns observed among the biogeographic areas or sites used in this study. However, one factor that does vary is predator diversity. Reefs of the Central Indo-Pacific are known to have greater overall diversity of fishes than those of the Eastern Indo-Pacific, and predator diversity has been shown to affect the grazing behavior of prey in at least one marine system [24]. Our metric of predation risk, however, incorporates differences in both predator abundance as well as predator diversity by including all observable predators. Other factors potentially affecting prey fish behavior, such as destructive fishing practices that alter benthic habitat and therefore shelter from predators (e.g., blast fishing; trawling) are not utilized in any of the sites used in this study. Depth, exposure, and other key characteristics of reefs were all standardized to the greatest extent possible and as such do not vary systematically among sites (see Materials and Methods). To our knowledge, no natural or anthropogenic factors other than fishing should therefore generate an *a priori* expectation matching our observed differences in prey fish behavior among reefs or biogeographic areas.

The limited number of reefs used in this study (eight reefs within four reef pairs) means that further study is needed before our findings can necessarily be generalized to reefs over larger scales and/or in different biogeographic regions. However, if the observed patterns were not general, or at least common, within the two biogeographic regions studied, the likelihood of concordance of patterns among all reef pairs in both regions would be low given that these reef pairs were haphazardly selected. These observed similarities among all reefs explored therefore suggest that some degree of generality of the patterns we document is likely.

This study is unique in its exploration of the restoration of prey foraging behavioral dynamics of reef ecosystems in response to conservation measures, in particular the temporal scale over which these responses can occur. To our knowledge, the only other studies to observe such effects in a natural, non-experimental system in which humans have caused predator loss (and predators have subsequently been allowed to recover) have been those on the human/wolf/elk interactions in the Rocky Mountains of North America [16], [25] where wolves were reintroduced after having been extirpated by human hunting many decades earlier. As such, this study provides novel insight into some of the behavioral consequences of management and conservation actions designed to mitigate human exploitation of marine systems, such as marine reserves or temporal fishery closures. In particular, we show that such conservation activities can induce behavioral responses that have been shown in other studies to have ecologically significant effects. Given the functional importance of parrotfish in particular in regulating algal communities on coral reefs, the shrinking of their grazing areas as marine reserves result in increased predator populations should be considered by reef managers. Specifically, our results suggest that as predator populations recover, managers may need to concomitantly aim to increase herbivore population sizes in order to maintain current grazing rates over all areas of a reef. Previous results have shown that fishing-induced changes in herbivore grazing behaviours have resulted in differences in macroalgal heterogeneity between fished and unfished reefs [14]. Reefs with large predator populations display a fine-scale mosaic of macroalgal biomass over the reef benthos, whereby areas of high macroalgal biomass are able to accumulate where predation risk is high and grazing is concentrated in ‘safer’ areas of the reef where risk is lower. Comparable reefs with depleted predator populations show more even grazing over the entire reef benthos and likewise greater homogeneity of algal biomass. If managers aim to maintain algal standing stock at current levels over all points of a reef's benthos, but concurrently aim to increase predator populations, our findings suggest that increasing herbivore population sizes may be a necessary prerequisite.

Within the context of the literature on behavioral trophic cascades, these findings provide a conceptual advance in demonstrating that, for at least the two species examined, the behavioral effects underlying these cascades (i.e., predator-induced changes in prey foraging behavior) are bidirectional and hence potentially reversible. Importantly, our findings show that by restoring heavily fished predator populations towards pre-disturbance levels, prey foraging behavioral dynamics have the capacity to rapidly return to states resembling those found in historically pristine systems.

## Materials and Methods

### Ethics statement

This study was approved by the University of California, Santa Barbara Institutional Animal Care and Use Committee (Protocol Approval # 403). No animals were harmed during the course of this study.

### Study systems

To separate the effects of food web alterations from biogeographic variation, we compared several pairs of reefs within different biogeographic realms where the members of each pair had different histories of exploitation. The study included sites within two areas of the Pacific Ocean, the Eastern Indo-Pacific (northern Line Islands, Republic of Kiribati and U.S.A.) and the Central Indo-Pacific (Great Barrier Reef (GBR), Australia; see [26]). We used two study sites in the Line Islands, Tabuaeran (heavily fished; Kiribati) and Palmyra (unfished, near-pristine; U.S.A.) Atolls. In the GBR, we examined three pairs of reefs. Each pair included one fished and one no-take marine reserve (i.e., recovering). We refer to these pairs as “GBR north,” “-mid,” and “-south,” however these terms denote only the relative geographic locations of the pairs; each of these reef pairs is located in the central/northern region within the context of the larger GBR. These reefs, and their respective histories of exploitation, are described in detail in Appendix S1.

### Piscivore surveys

We conducted surveys of piscivorous fish biomass at each reef to serve two purposes. First, these data were used to determine if differences in human fishing pressure had indeed resulted in relative differences in piscivorous fish biomass between paired reefs. Secondly, these data served as a measure of chronic predation risk to which non-predatory (i.e., prey) fishes are exposed. Chronic predation risk is defined as risk integrated over space (i.e., entire reefs) and time, or the ambient predation risk found at a given reef [13]. These surveys consisted of replicate 30×2 m belt transects in which all fishes known from the primary literature [27], [28] and/or a standardized global database [29] to consume other fishes were censused. We included only individuals of these species greater than 10 cm TL, and therefore potentially capable of consuming the smallest adult individuals of the study's smaller focal prey species (the blackbar damselfish, *Plectroglyphidodon dickii*). Many predatory species with large home ranges will result in high variability and low confidence of absolute population size estimates at this scale of measurement. However, our objective was simply to generate relative estimates of predator abundance among our sites, for which this sampling scale is appropriate. Importantly, predator biomass has been shown to be a more reliable metric of detecting fishing effects on coral reefs [30] especially where larger, less abundant fish such as predators are primarily targeted. Additionally, our biomass-based metric has been explicitly compared to abundance-based metrics (for both chronic and acute predation risk) [13] with nearly identical results. Abundance-based metrics must incorporate a size threshold in order to be meaningful representations of predation risk for prey (otherwise, for example, an individual predator that is smaller than its prey counterpart could be counted). Biomass-based metrics eliminate the need for such subjective delineations because they are inherently weighted by predator size, and presumably thus threat level for potential prey. Further details of piscivore surveys can be found in Appendix S1.

### Behavioral observations

To quantify prey excursions, which represent a trade-off between competing demands of food acquisition and predator avoidance, we observed adult individuals of two common, non-predatory, primarily herbivorous fish species at all reefs. The blackbar damselfish (*Plectroglyphidodon dickii*), a small, site-attached damselfish, and the bullethead parrotfish (*Chlorurus sordidus*), a larger, mobile parrotfish, were chosen because they are relatively abundant at all study reefs, they represent different herbivore functional groups, and they exhibit distinctly different behavioral characteristics. All observations were conducted for five-minute bouts, with an approximately three-minute habituation period for each focal individual prior to observation. These behavioral observations followed the protocol outlined in Madin et al. [13]. The specific metric used to quantify prey excursion size varies slightly by species because of the different behavioral characteristics of the species. In particular, excursions of the bullethead parrotfish are measured simply as the area covered by each focal fish during the 5-minute observation period, with excursion size for this species in the Central Indo-Pacific sites estimated from rate of movement (i.e., excursion speed; see Appendix 1 and Fig. S1 for details). Conversely, for the blackbar damselfish it was necessary to take into account the area of the focal fish's home coral colony (their central refugia, from which all excursions radiate). Incorporating shelter size into the metric was necessary because the size of the focal fish's home shelter determines the area of reef that the focal fish can safely travel over without incurring significant risk of being consumed by a predator. Therefore, larger home coral colonies lead to inflated excursion sizes of this species, and it was necessary to scale this species' excursion area by that of its home shelter.

In addition to observing prey behavior, we simultaneously generated a measure of acute risk to which each focal prey individual was exposed while under observation. Acute risk is the immediate risk that a given focal individual experiences during the period over which its behavior is observed, and as such it is measured on the basis of the individual predators that the focal individual encountered during this time [13]. Specifically, all piscivores approaching within a defined range of the focal individual (a 3 m^3^ cube for *P. dickii* and a 3 m radius sphere for *C. sordidus*) were censused during each five-minute observation period. For the site-attached *P. dickii*, the amount of time that each predator spent in the immediate area surrounding the focal individual was also measured. For this species, acute risk was calculated as the biomass of predators multiplied by the duration of their visit that each focal prey fish encountered over the five-minute observation period. Acute risk was calculated for the mobile species (*C. sordidus*) as the biomass of predators that each focal prey fish encountered over the five-minute observation period. Using the above acute risk metric for *P. dickii*, differences between both acute risk and foraging excursion areas were then calculated between paired reefs for this species. Differences in predation risk (piscivore biomass * duration of visit) between reefs was calculated as:
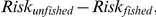
with the order of the terms reflecting the expected direction of change (i.e., more predators found in unfished than fished reefs within each pair). Similarly, prey excursion size (ratio of excursion area to shelter area) was calculated as
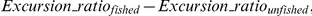
where the order of the terms again reflects the expected direction of change (i.e., greater excursions in fished than unfished reefs within each pair). Further details on behavioral observations and all data analyses can be found in Appendix S1.

## Supporting Information

Figure S1 **Relationship between excursion area (m^2^) and rate of movement (cm/s) for** ***C. sordidus*** **.** Data are from three atolls within the Line Islands (Palmyra, Tabuaeran, Kiritimati).
(TIFF)

Figure S2 **Piscivores encountered by** ***P. dickii*** **in relation to reef protection status.** Points are means (±SE).
(TIFF)

Figure S3 **Density of competitors for focal species within Central Indo-Pacific reef pairs.** Upper panel (a) is blackbar damselfish (*P. dickii*); lower panel (b) is bullethead parrotfish (*C. sordidus*). Bars are means (±SE).
(TIFF)

Appendix S1 **Methodological details.**
(DOC)
