# Supplementary figures and images for: Do Behavioral Foraging Responses of Prey to Predators Function Similarly in Restored and Pristine Foodwebs?
Source: PLoS One. 2012 Mar 5;7(3):e32390. doi: 10.1371/journal.pone.0032390 (PMC3293809; doi:10.1371/journal.pone.0032390)

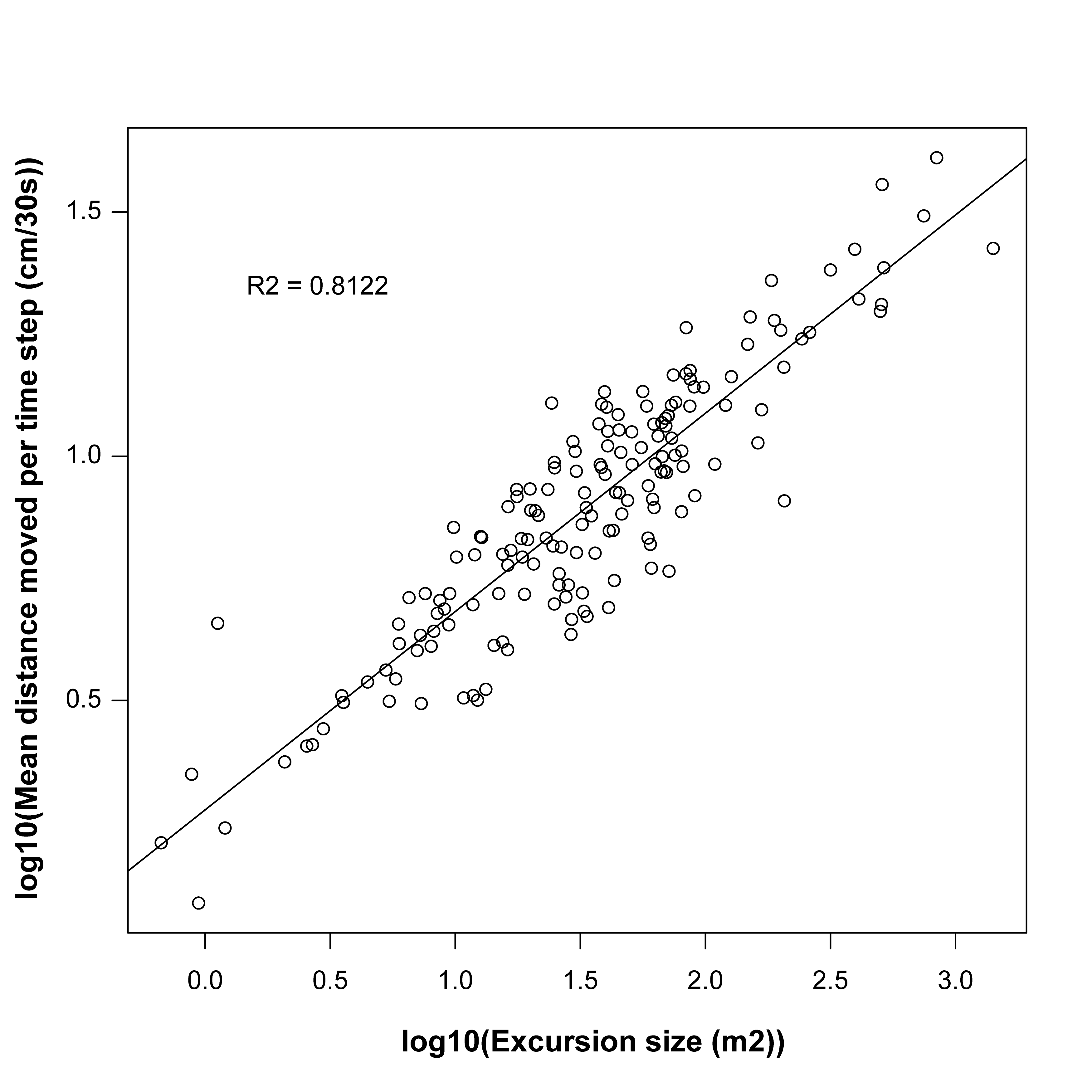

Supplement: Figure S1 — Relationship between excursion area (m2) and rate of movement (cm/s) for C. sordidus . Data are from three atolls within the Line Islands (Palmyra, Tabuaeran, Kiritimati). (TIFF) [file pone.0032390.s001.tif]

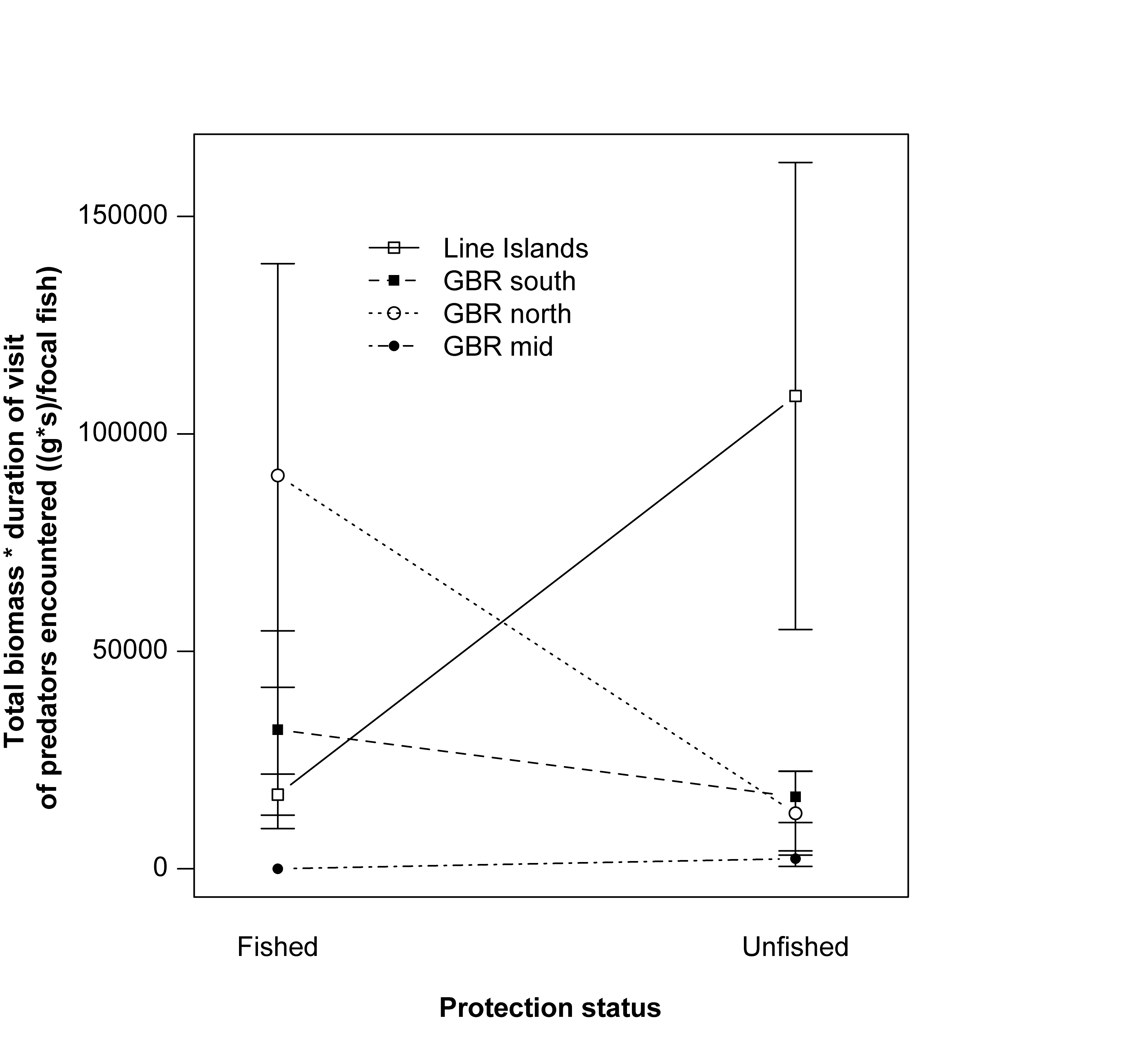

Supplement: Figure S2 — Piscivores encountered by P. dickii in relation to reef protection status. Points are means (±SE). (TIFF) [file pone.0032390.s002.tif]

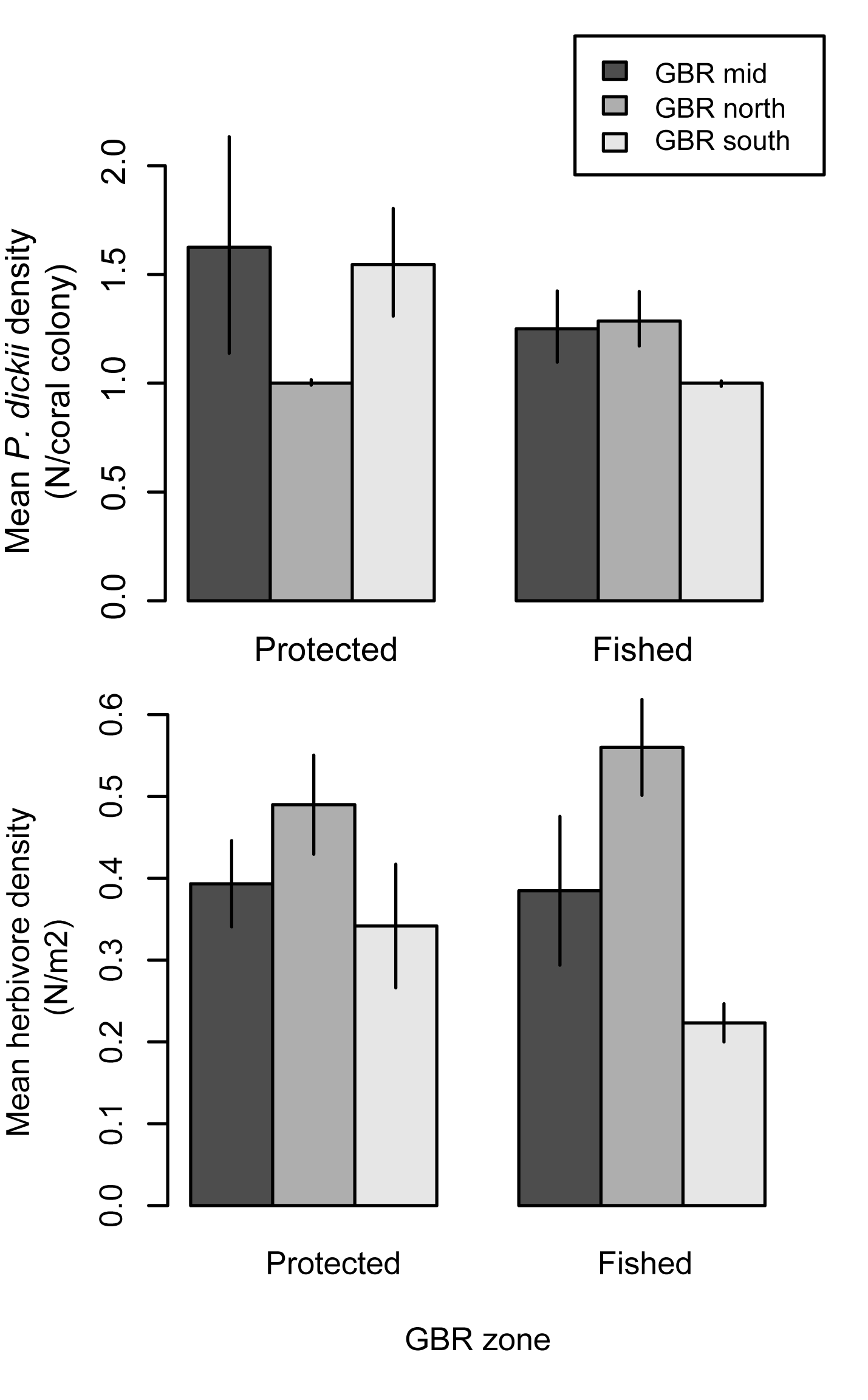

Supplement: Figure S3 — Density of competitors for focal species within Central Indo-Pacific reef pairs. Upper panel (a) is blackbar damselfish (P. dickii); lower panel (b) is bullethead parrotfish (C. sordidus). Bars are means (±SE). (TIFF) [file pone.0032390.s003.tif]
