# Supplementary material for: Do Behavioral Foraging Responses of Prey to Predators Function Similarly in Restored and Pristine Foodwebs?
Source: PLoS One. 2012 Mar 5;7(3):e32390. doi: 10.1371/journal.pone.0032390 (PMC3293809; doi:10.1371/journal.pone.0032390)
Supplement: Appendix S1 — Methodological details. (DOC) [file pone.0032390.s004.doc]

Appendix S1. Methodological details.

*Study system*

Situated with the outer Line Islands of the Republic of Kiribati, Tabuaeran Atoll has sustained substantial, permanent human settlement since approximately 1988, when a resettlement program began to move I-Kiribati (citizens of the Republic of Kiribati) from the country’s densely populated Gilbert Islands archipelago to their remote and essentially unpopulated northern Line Islands archipelago in an effort to reduce overpopulation in the former islands (9). Prior to this time, only small settlements had existed periodically on this atoll since the first copra (coconut meat) plantations were established there in the 1850s (9). Human occupation of this atoll has resulted in essentially unregulated fishing pressure, primarily for subsistence purposes. By contrast, Palmyra Atoll, an unincorporated territory of the U.S.A., has never sustained permanent human settlement (other than a brief military occupation during WWII). Since 2001, Palmyra has further been legally protected from all extractive fishing by the U.S. Fish and Wildlife Service. Additional details of the respective histories of fishing pressure on these reefs is documented elsewhere (8, 9), and further details on the sites used within these reefs are described in detail in Madin et al. (13).

On the Great Barrier Reef, we examined three pairs of reefs. Each pair included one fished and one no-take marine reserve (i.e., a currently unfished reef). From north to south, the reef pairs are: Sandbank #5 (fished) and Reef #13-125 (unfished, as part of Marine National Park 13-1017), hereafter referred to as “GBR north”; Hicks (fished) and Day (unfished) Reefs, hereafter referred to as “GBR mid”; and Arlington (fished) and Hastings (unfished) Reefs, hereafter referred to as “GBR south”. From the early- to mid-1990s until 2004, each of these reefs was located within “Habitat Protection Zones” of the GBR Marine Park except Hastings Reef, which was located within a “Marine National Park Zone”. Within “Habitat Protection Zones”, all types of fishing are allowed (a few via individual permits) with the exception of trawling. In 2004, the GBR Marine Park was rezoned to include a greater proportion of each marine habitat type in no-take reserves. Since that time, the “fished” reefs in each of the two northerly pairs have remained within “Habitat Protection Zones,” while the “unfished” reefs of these pairs are now within “Marine National Park Zones”. In this zone, no fishing is allowed other than traditional use of marine resources (i.e., indigenous fishing for traditional target species using low-tech gear). Indigenous fishing is likely extremely limited on these study reefs, given their substantial distance from coastal settlements (60-90 nautical miles from the nearest coastline). Therefore, since the early 1990s, the unfished reef within the pair “GBR south” has been protected from essentially all extractive uses, while those in the pairs “GBR mid” and “GBR north” have been protected only since 2004.

*Piscivore surveys*

For surveys of piscivorous fish biomass at each reef, observers recorded the species identity and total length (TL) of each individual encountered, and publicly-available length-weight conversions (20) were later used to calculate the biomass of each individual. The number of 30 x 2m m belt transects within sites differs between the two biogeographic realms: in the Central Indo-Pacific (GBR), ten transects were conducted per reef, whereas in the Eastern Indo-Pacific (Line Islands) 40 and 110 transects were conducted at the fished and unfished reefs, respectively. The Eastern Indo-Pacific reefs were surveyed more intensively as part of a longer-term study of reef dynamics. Surveys were conducted by pairs of observers, with the primary observer (EM) remaining consistent throughout all surveys in both biogeographic realms. All surveys were conducted between 2-10m depth using a mix of snorkel and SCUBA apparatus, with both methods employed at all sites. All GBR sites were located on the protected (i.e., non-ocean facing) back reef edge, while sites at both the Line Islands sites were conducted in a mix of exposed and protected reefs, in order to standardize the level of physical exposure regimes within reef pairs. Transects within sites were located so as to minimize any differences in reef topography and dominant habitat type among both transects and sites, but within these constraints were placed haphazardly. All surveys were thus conducted in areas dominated by live coral and other live benthic substrate (i.e., as opposed to sand- or rubble-dominated zones).

In terms of the acute predation risk metric, the size of the areas surrounding focal prey individuals (i.e., a 3 m^3^ cube for *P. dickii* and a 3 m radius sphere for *C. sordidus*) within which predators were counted were chosen based on the observation that beyond them, individuals did not generally appear to react to the presence of predators. It was thus assumed that the perceived risk to prey individuals was minimal beyond these distances. The different methods of predator counts (i.e., cube versus sphere) reflect logistical constraints imposed by the dominant behavioral types of the two focal species. Specifically, a stationary cube delimited by weighted markers could be used for the site-attached *P. dickii*, whereas this was not practical for the mobile *C. sordidus*. In the latter case, a sphere enabled more reliable counts of predators that focal individuals encountered as they moved over the reef.

Piscivore biomass has been shown to be a robust metric of predation risk, in particular when compared with metrics based on piscivore abundance filtered by a size threshold (13). A biomass-based rather than abundance-based metric of predation risk was utilized because biomass incorporates the sizes, in addition to abundances, of the predators that prey fishes encounter, a likely important factor in determining perceived predation risk. This method is particularly useful because it requires no subjective assumptions to be made by the researcher regarding which predators to include or exclude (e.g., as are necessary when using a size threshold or pairwise threat rankings) and it can be calculated based on commonly collected, easily obtainable data (13). However, this metric necessarily counts some piscivores that pose no threat to a given prey, for example in cases where a piscivore is smaller than a focal prey individual. This characteristic of the metric renders it conservative; including non-threatening predators should decrease the likelihood of detecting an effect of predators on anti-predator behavior in prey.

*Herbivore surveys*

In order to determine if local competitor density could be responsible for reef-scale behavioural patterns, these densities were quantified for the relevant suite of potential competitors predicted to influence focal prey excursion size. The mobile species *C. sordidus* encounters both territorial and non-territorial benthic herbivores continually as it moves over the reef to feed, the former of which actively deters grazing within their territories and both of which contribute to crowding. Therefore, the suite of potential competitors predicted to influence excursion size for this focal species includes all benthic herbivores. Benthic herbivore abundance was quantified along the piscivore transects described above at each reef. For the site-attached, central place foraging species *P. dickii*, as interspecific group size within a given colony increases, so too should the competition for resources surrounding the home coral colony and thus the need to move farther from this central place to acquire a given amount of food. Therefore, the suite of potential competitors predicted to influence its excursion size includes conspecific individuals inhabiting each focal individual’s home coral colony. The number of conspecifics per home coral colony for each focal individual’s home colony was quantified immediately prior to focal individual behavioural observations.

*Behavioral observations*

Within each reef, observations of prey excursions were conducted in the same vicinity as the piscivore surveys and thus variability in exposure, depth, topography, and habitat type was minimized. Within these areas, focal individuals were chosen haphazardly as they were encountered. Similar distributions of focal individual body sizes (and hence vulnerability to predation) were used for both species within all reef pairs (two-sample Kolmogorov-Smirnov test for *C. sordidus* (GBR north *P* = 0.401; GBR mid *P* = 1; GBR south *P* = 0.759; Line Islands *P* = 0.806) and *P. dickii* (GBR north *P* = 0.229; GBR mid *P* = 0.627; GBR south *P =* 0.0634)) with the exception of *P. dickii* in the Line Islands pair (*P* = 0.012). In this case, however, the mean body size of the focal individuals was nearly equivalent (Line Islands fished site = 7.01 cm TL; Line Islands unfished site = 6.02 cm TL), indicating that this factor is not likely to be responsible for any differences the excursion sizes between these sites.

All observations were conducted between 0800 – 1700 hours, with roughly equivalent distributions of observations throughout this period for both focal species within all reef pairs (Welch’s two-sample T-test for *C. sordidus* (GBR mid *P* = 0.822; GBR south *P* = 0.854; Line Islands *P* = 0.165) and *P. dickii* (GBR north *P* = 0.365; GBR mid *P* = 0.711; GBR south *P* = 0.332; Line Islands *P* = 0.117)) with the exception of *C. sordidus* in the GBR north pair (*P* = 0.001). In this case, however, the mean time of day of the observations was nearly equivalent (GBR north fished site = 12:12; GBR north unfished site = 12:48), indicating that this factor is similarly unlikely to appreciably influence the magnitude of excursion behaviors observed. Observers remained between 3 – 5 m from focal individuals at all times during the five-minute observation period, except when the fish under observation swam towards the observer. In such cases, which occurred at all reefs in approximately the same frequency, the observer remained motionless until the fish was again at least 3 m away. Observation of an individual was discontinued and data discarded if either the fish was lost or showed any indication of observer interference (e.g., hiding; flight), although the latter (apparent observer interference) was never observed. Observations were also discontinued if water visibility fell to < 5 m.

For the mobile focal prey species (the parrotfish *Chlorurus sordidus*), only initial phase individuals of *C. sordidus* were included in order to standardize observations and avoid incorporating known differences in behavior due to developmental phase (i.e., juvenile vs. initial vs. terminal phases). Excursion size was determined by dropping numbered subsurface markers (weights attached to corks with ~25 cm of flagging tape) at regular (30 second) intervals along the offset path of the focal fish over the observation period. Markers were placed approximately 5 m downstream of the focal individual, resulting in a record of the path that the individual took over the observation period offset from the actual path by 5 m. At the conclusion of each 5-minute observation period, the distances between markers was measured sequentially in the order in which they were dropped. These distances were then summed and divided by the total number of seconds (300) for which the fish was observed to generate the average distance moved per second by the individual (excursion speed). In the Eastern Indo-Pacific, the area of reef covered by the individual (excursion area) was then measured by estimating the area of the smallest ellipse that included all the distance markers. The area of the ellipse (Π*ab*) was approximated from the distance between the two most widely separated markers (major axis – 2a) and the two most widely separated markers perpendicular to that axis (minor axis – 2*b*). Since rate of movement (excursion speed) and excursion area were highly correlated for 167 *C. sordidus* individuals (Fig. S1), only speed was estimated for the Central Indo-Pacific sites and excursion area was estimated using the Eastern Indo-Pacific coefficients.

For the site-attached omnivore (the primarily herbivorous damselfish *Plectroglyphidodon dickii*), excursion area was determined by dividing the reef substrate into four quadrants whose origin was at the center of the focal individual’s home coral colony, then observing the farthest distance in each quadrant that the individual ventured from the home coral colony during the 5-minute observation period. At the conclusion of the period, four markers were placed at these points within each of the quadrants, and the distances between opposing pairs measured. These orthogonal distances were then used to calculate the excursion area as described above. In order to standardize by the area of shelter provided by the home coral colony within this area, the area of the coral colony was measured (using its longest and perpendicular axes as the major and minor axes, respectively) and the ratio of excursion area to shelter area calculated. Shelter coral taxa was similar across all sites and consisted primarily of *Pocillopora spp.* and tabular *Acropora spp.*, with the exception of finely branching *Acropora spp.* which were only found at Palmyra Atoll. Because of the unique morphology of these latter refuges, individuals inhabiting these corals were excluded from analyses.

*Data analysis*

As described by Madin et al. (13), predation risk often affects maximum excursion distances more than average excursion distances. This is because predation risk may constrain some actions prey will take far more than others. Prey under high risk must balance limiting risky behaviors with maintaining other necessary functions (31), whereas prey under low risk may freely engage in otherwise risky behaviors or may choose to limit their excursions for other reasons (e.g., ample food supply in a particular location, etc.). Thus, risk should constrain the upper bound of foraging excursions, in addition to or in place of affecting the slope of the regression. In some cases, the upper bound of a distribution may decline, indicative of declining maximum excursion sizes, even when the mean relationship between risk and excursion distance is not negative. For this reason, we used a linear regression model in which variance in the excursion size was allowed to vary independently and as a function of acute predation risk. Thus the slopes of the upper bound (95% prediction interval) could be estimated separately from the slope of the regression. These slopes were calculated for both species in both major areas and tested for significance using 1000 randomizations without replacement following the method outlined in Madin et al. (13). All significance tests were conducted at α = 0.05. All data analyses were conducted using R version 2.8.1 (R Development Core32).

When used to determine the effect of human fishing pressure on piscivorous fish biomass, piscivore biomass data were analyzed by two-way ANOVA with status (fished vs. protected) and region (GBR north/mid/south and Line Islands) as factors with sites as replicates. Because of the large home range sizes and low abundances of larger piscivores (e.g., reef sharks, larger carangids and lutjanids, etc.), our surveys were designed to provide accurate relative, but not necessarily absolute, estimates of their abundances within and among reef pairs. When used as a measure of chronic risk for prey species, these data were log_10_ transformed (with addition of a constant of 1 to all observations to account for transects with zero piscivore biomass values).

For comparisons of prey behavior between reef pairs and regions (i.e., under chronic predation risk), behavioral excursion data were analyzed by two-way ANOVA with status (fished vs. protected) and region (GBR north/mid/south and Line Islands) as factors, with sites as replicates.

A shifted log transform with a shift parameter of α (a constant) was used to incorporate data on focal prey species’ excursions in which no predators were encountered using the acute risk metric (i.e., predator biomass = 0), and those where prey excursions had a value of < 1, into upper bound and regression analyses. This method was selected as preferable to the alternative of treating x = 0 values as missing, which results in a more normalized distribution of residuals, because it allows extreme values of both predation risk (x) and prey behavior (y) to be incorporated into analyses. The α value used for each species’ predator biomass values was determined by the mean body size of each focal prey species and thus its vulnerability to predators of different body (and hence mouth gape) sizes. These values were chosen to represent realistic predator biomass values indicative of a predator that would be at or just below the lower end of the range of possible threats posed by predators for each prey species and are 300g (*C. sordidus*) and 400 (representing an 80g predator in the prey’s vicinity for 5 seconds; *P. dickii*). Sensitivity analyses were performed for each species to determine the effect that the **α** value chosen for predator biomass had on the regression and upper bound statistics. An α of 1 was similarly added to all prey excursion size values for regression and upper bound analyses for the three species (*C. sordidus; A. nigricans; P. dickii*) whose minimum excursion value was < 1 and thus led to a negative value when log_10_-transformed.
